# Does orthodontic treatment need have an impact on oral health-related quality of life?

**DOI:** 10.1007/s00056-022-00438-y

**Published:** 2023-02-01

**Authors:** Katrin Bekes, Kathrin Kuhr, Cristiana Ohm, Nicolas Frenzel Baudisch, Andreas Rainer Jordan

**Affiliations:** 1 Department of Pediatric Dentistry, Medical University Vienna, University Clinic of Dentistry, Sensengasse 2a, 1090 Vienna, Austria; 2 Institute of German Dentists, Universitätsstr. 73, 50931 Cologne, Germany

**Keywords:** Survey, Oral Health Impact Profile (OHIP), Malocclusion, Index of Orthodontic Treatment Need (IOTN), Sixth German Oral Health Study (DMS 6), Erhebung, Oral Health Impact Profile (OHIP), Malokklusion, Index der kieferorthopädischen Behandlungsnotwendigkeit (IOTN), Sechste Deutsche Mundgesundheitsstudie (DMS 6)

## Abstract

**Objectives:**

The aims of this study were to determine the frequency of oral health-related quality of life (OHRQoL) impairment in a national representative sample of 8 to 9 year olds in Germany and to evaluate the impact of orthodontic treatment need.

**Methods:**

Data were collected in the Sixth German Oral Health Study (Sechste Deutsche Mundgesundheitsstudie, DMS 6) and subjects were sampled using a multistage sampling technique. OHRQoL was measured with a modified version of the 5‑item Oral Health Impact Profile (OHIP-5) which was administered in a computer-assisted personal interview. Children were also examined for malocclusion and orthodontic treatment need.

**Results:**

In all, 1892 children aged 8–9 years were invited to take part. Finally, data of 705 children (48.6% female) could be included in the analysis. The OHIP‑5 mean was 1.3 (±2.0). There was no relevant influence from age and gender on the OHIP‑5 summary scores (r < 0.10), but the summary scores differed when analyzed separately regarding orthodontic treatment need or no orthodontic treatment need (1.5 ± 2.0 vs. 1.2 ± 1.9, *p* = 0.020). Nevertheless, the level appears to be low.

**Conclusions:**

Malocclusions with orthodontic treatment need have an influence on OHRQoL.

## Abstract (de)

**Zielsetzung:**

Ziele dieser Studie waren die Ermittlung der Häufigkeit von Beeinträchtigungen der mundgesundheitsbezogenen Lebensqualität (OHRQoL) in einer national repräsentativen Stichprobe von 8‑ bis 9‑Jährigen in Deutschland und die Bewertung des Einflusses des kieferorthopädischen Behandlungsbedarfs.

**Methoden:**

Im Rahmen der Sechsten Deutschen Mundgesundheitsstudie (DMS 6) wurden die Daten erhoben, die Probanden wurden anhand einer mehrstufigen Stichprobenmethode ausgewählt. Die OHRQoL wurde mit einer modifizierten Version des 5‑Item Oral Health Impact Profile (OHIP-5) erhoben, dafür wurden computergestützte persönliche Interviews durchgeführt. Die Kinder wurden auch auf Zahnfehlstellungen und kieferorthopädischen Behandlungsbedarf untersucht.

**Ergebnisse:**

Insgesamt wurden 1892 Kinder im Alter von 8–9 Jahren zur Teilnahme eingeladen. Letztendlich konnten die Daten von 705 Kindern (48,6 % weiblich) in die Analyse einbezogen werden. Der OHIP-5-Mittelwert lag bei 1,3 (±2,0). Es gab keinen relevanten Einfluss von Alter und Geschlecht auf die OHIP-5-Summenscores (r < 0,10), aber die Summenscores unterschieden sich, wenn sie getrennt nach kieferorthopädischem Behandlungsbedarf bzw. keinem kieferorthopädischen Behandlungsbedarf analysiert wurden (1,5 ± 2,0 vs. 1,2 ± 1,9, *p* = 0,020). Dennoch scheint das Niveau niedrig zu sein.

**Schlussfolgerungen:**

Zahnfehlstellungen mit kieferorthopädischem Behandlungsbedarf haben Einfluss auf die OHRQoL.

## Introduction

Malocclusion is one of the most important and prevalent oral health problems worldwide [1]. It is defined as a developmental condition with a deflection from the normal relation or alignment of the teeth to other teeth in the same arch and/or to the teeth in the opposing arch [2]. It can vary from minor esthetic to severe. According to a recent review [3], the global distributions of Angle class I, class II, and class III malocclusions in permanent teeth are estimated to be 75, 20, and 6%, respectively. Vertical malocclusions, such as deep overbite and open bite affect can be found in around 22% and 5% of the cases, and posterior crossbite can be observed in 9%. Malformation of the dentition can be accompanied with physical (e.g., chewing, swallowing, and speaking skills) and psychological challenges (e.g., esthetics) and can therefore have an impact on a person’s daily life [4].

An objective understanding of the patient’s opinion regarding his/her health can be derived by patient-reported outcomes which have also received increasing attention in recent years in pediatric dentistry as they support patient-centered care and clinical indicators alone do not reveal the full impact of oral conditions on the psychosocial well-being of a patient [5]. The most important dPRO is oral health-related quality of life (OHRQoL), which “reflects people’s perspective on their oral health status including eating, sleeping and engaging in social interaction; their self-esteem; and their satisfaction with respect to their oral health” [6]. OHRQoL can be assessed using questionnaires (dental patient reported measures [dPROMS]). However, some issues arise when measuring OHRQoL in younger patients due to their different phases of physical cognitive, emotional, social and language development, as oral health and health cognition are considered age-dependent [7, 8]. Therefore, several dPROMs exist for children as well as for adults [5, 9], taking into account different age groups. In adults, the Oral Health Impact Profile (OHIP) is the most widely used and accepted instrument internationally [10, 11]. Currently, the short version of the OHIP, the 5‑item OHIP (OHIP-5) is recommended for oral health impact measurement [12]. Originally, this instrument was not designed for children or adolescents. However, it has already been applied in some studies to evaluate OHRQoL in younger age groups [13, 14]. Moreover, its validity and reliability were found satisfactory [15]. Therefore, it can be assumed that the OHIP is also applicable for school-children and adolescents.

Until now, there are no national representative data available for OHRQoL in German 8–9 year olds in general as well as regarding to orthodontic treatment need.

Therefore, the aims of this study were to determine the frequency of oral health-related quality of life (OHRQoL) impairment in a national representative sample of 8–9 year olds in Germany and to evaluate the impact of orthodontic treatment need using the 5‑item version of the OHIP. The study is part of the Sixth Oral Health Study (Sechste Deutsche Mundgesundheitsstudie, DMS 6), in particular the orthodontic module (module kfo(“Kieferorthopädie”)-6.1).

## Materials and methods

### Subjects

The study population represents a nationwide population-representative collective of children aged 8–9 years in Germany. The sampling was stratified according to the characteristics of the federal states and bik region size classes^999^. For this study, a random sample of 16 municipalities was selected from the 90 municipalities of the Fifth Oral Health Study (DMS V), stratified according to federal states. In addition to the federal state as a stratification characteristic, the selection also took into account a simplified variant of the bik region size classes. If the population in the selected sample municipalities was not sufficiently large, so-called synthetic points were formed from several surrounding municipalities. In a second stage, the target persons were chosen at random. This was based on the personal registers of the residents’ registration offices.

Sample size was calculated regarding the primary aim of the study project, which was to assess the prevalence of malocclusions in 8‑ and 9‑year-old children in Germany. Thereby, the number of cases should be sufficient to estimate the current prevalence of malocclusions in Germany (module kfo‑6.1) as well as to have sufficient study participants for the planned resurvey in 2030 (module kfo‑6.2). The basis for the calculations on the expected number of cases in module kfo‑6.2 was the available data set of the comparable cohort of 12 year olds from the DMS V. It was assumed that 95% of the study participants from module kfo‑6.1 met the inclusion criteria for inclusion in the panel. With an annual lost-to-follow-up rate of 3% and a response rate of 70%, a sample size of 670 study participants in 2021 resulted in an estimated gross case number of 483 available persons in 2030. In view of the uncertainties with regard to (a) possible impairments of the field work by political measures in connection with the coronavirus disease 2019 (COVID 19) pandemic and (b) the lack of empirical values on the willingness of study participants to participate in a pandemic situation, a comparatively high gross number of addresses was chosen for safety with a so-called triple translation.

The children were invited to the study center with their parents. There, a personal interview was conducted first, followed by tooth brushing and a dental examination. Beside other questions focusing on group prophylaxis and oral hygiene behavior, the computer-assisted personal interview (CAPI) included the assessment of OHRQoL using the OHIP‑5. The children were clinically examined by one dentist who was trained and calibrated. Dental examination included orthodontic clinical findings, orthodontic intraoral scan, presence of caries and restorations, plaque and gingival recession, and gingival bleeding.

Approval for this study was obtained from the ethics committee of the local University Review Board (University of Witten Herdecke; No. 113/2020).

### Modified OHIP-5 for children

The 5‑item OHIP questionnaire (OHIP-5) is an ultrashort version of the original 49-item OHIP which was introduced in Germany and developed using best subset regression [12]. The instrument contains only 10% of the items but captures about 90% of the score information compared with its original version [16]. The 5 items of the OHIP‑5 focus on functional limitation, pain, psychological discomfort, physical disability, and handicap. Questions ask about the frequency of events during the last week. Responses are made on an ordinal scale from 0 to 4 (0 = never, 1 = hardly ever, 2 = occasionally, 3 = fairly often, and 4 = very often). Higher scores refer to a worse OHRQoL status. Summing the response codes for the questionnaire items generates an overall OHIP score. The instrument’s summary score ranges from 0–20. A summary score of zero indicates the absence of any problems, and a higher OHIP score represents more impaired OHRQoL.

In the present study, a slightly modified German version of the OHIP‑5 was used. First, the formal form of “you/Sie” in German was substituted with an informal form “you/Du”. Second, the term “teeth, mouth or dentures” in each question was replaced by “teeth, mouth, dentures or braces”. Third, the question “Have you had difficulty doing your usual jobs because of problems with your teeth, mouth or dentures?” was supplemented with explanations: “Have you had difficulty doing your usual jobs *(e.g., with your family, at school, with your friends)* because of problems with your teeth, mouth, dentures or braces?”

In addition to the 5 items, the children were also asked for a global rating of the oral health and the overall well-being. These global ratings had a 5-point response format (excellent, very good, good, moderate, poor).

When starting the interview about OHRQoL with the child, the interviewer gave a short introduction. “Now I have a few questions for you about problems with your teeth. Here are the questions (questionnaire was shown and handed out). I will read them and you can read along. At the bottom of the page you will see a grey beam that is getting lighter and lighter. In it are the words ‘very often’, ‘often’, ‘occasionally’, ‘hardly ever’ and ‘never’. I’m going to ask you how often you had certain problems with your teeth. Please answer with the words from the grey beam, either ‘very often’, ‘often’, ‘occasionally’, ‘hardly ever’ or ‘never’.”

## Results

### Study population

A total of 1892 children were initially contacted and invited to take part in the study. After the exclusion of quality neutral defaults and systematic failures, 705 study participants (48.6% female) could be included (response rate 40.6%) for data analysis: 49.4% (*N* = 348) of the children were 8 years old, 50.4% (*N* = 357) were 9 years of age.

### Modified version of the OHIP-5

All 5 items of the OHIP were considered comprehensible. The children were able to answer all questions. When approached as to whether they had questions or needed assistance, the children indicated that they understood all questions.

### Oral health-related quality of life

Half of the study participants (50.6%) did not show any impairment of OHRQoL. The mean OHIP‑5 score was 1.3 (±2.0; range 0–14; Table 1). Detailed answers of the five questions can be found in Table 2. There was no relevant influence from age and gender on the OHIP‑5 summary scores (r < 0.10). The most important problem reported by the children (OHIP answer categories ‘often’ and ‘very often’) was ‘painful aching’ (3.2%; Table 2).

**Table 1:** OHIP‑5 mean scores for the whole national sample (*N* = 705) Mittlere OHIP-5-Scores für die gesamte nationale Stichprobe (*n* = 705)

OHIP	Mean (±SD)	95% CI	Median	Min–Max
*Total*	*1.3 (±2.0)*	*1.3 (1.2–1.5)*	*0 [0–2]*	*0–14*
OHIP 1	0.3 (±0.8)	0.3 (0.3–0.4)	0 [0–0]	0–4
OHIP 2	0.4 (±0.8)	0.4 (0.3–0.4)	0 [0–0]	0–4
OHIP 3	0.4 (±0.8)	0.4 (0.4–0.5)	0 [0–1]	0–4
OHIP 4	0.1 (±0.4)	0.1 (0.1–0.1)	0 [0–0]	0–3
OHIP 5	0.1 (±0.4)	0.1 (0.1–0.1)	0 [0–0]	0–3

*OHIP‑5* 5-item Oral Health Impact Profile, *CI* confidence interval, *SD* standard deviation

**Table 2:** Detailed answers of each OHIP item in the whole national sample (*N* = 705) Detaillierte Antworten zu jedem Item des OHIP in der gesamten nationalen Stichprobe (*n* = 705)

OHIP	Answer category	% (95% CI)	*N*
OHIP 1 Difficulty chewing any foods	Never	80.4 (77.3–83.2)	566
	Hardly ever	10.3 (8.2–12.7)	72
	Sometimes	7.0 (5.3–9.1)	49
	Often	1.4 (0.7–2.5)	10
	Very often	1.0 (0.5–2.0)	7
OHIP 2 Painful aching	Never	78.7 (75.5–81.6)	555
	Hardly ever	10.5 (8.4–12.9)	74
	Sometimes	7.7 (5.9–9.9)	54
	Often	1.5 (0.8–2.7)	10
	Very often	1.7 (1.0–2.9)	12
OHIP 3 Felt uncomfortable about the appearance	Never	74.1 (70.7–77.2)	521
	Hardly ever	11.9 (9.7–14.5)	84
	Sometimes	11.5 (9.3–14.0)	81
	Often	1.5 (0.8–2.7)	11
	Very often	1.1 (0.5–2.1)	8
OHIP 4 Less flavor in food	Never	92.0 (89.8–93.8)	645
	Hardly ever	5.9 (4.4–7.9)	41
	Sometimes	1.5 (0.8–2.7)	10
	Often	0.6 (0.2–1.5)	4
	Very often	0.0 (0.0–0.5)	0
OHIP 5 Difficulty doing usual jobs	Never	92.1 (89.8–93.9)	648
	Hardly ever	5.3 (3.9–7.2)	37
	Sometimes	2.2 (1.3–3.5)	15
	Often	0.4 (0.2–1.3)	3
	Very often	0.0 (0.0–0.5)	0

*OHIP* Oral Health Impact Profile, *CI* confidence interval

Regarding orthodontic treatment need, it could be observed that children in need for treatment showed a significant higher OHIP score (1.5 ± 2.0) than children having no need for treatment (1.2 ± 1.9; *p* = 0.020). However, the level appears to be low. The OHIP item focusing on “difficulty chewing food” also showed a significant difference in mean scores (0.4 ± 0.8 vs. 0.3 ± 0.8; *p* = 0.011; Table 3).

**Table 3:** OHIP mean scores in the national sample regarding orthodontic treatment need Mittlere OHIP-Scores in der nationalen Stichprobe hinsichtlich kieferorthopädischer Behandlungen

	Orthodontic treatment need (*N* = 285)		No orthodontic treatment need (*N* = 420)		
OHIP	Mean (±SD)	Median	Mean (±SD)	Median	*P*–value
OHIP 1	0.4 ± 0.8	0 [0–0]	0.3 ± 0.7	0 [0–0]	0.011
OHIP 2	0.4 ± 0.9	0 [0–0]	0.3 ± 0.8	0 [0–0]	0.084
OHIP 3	0.4 ± 0.8	0 [0–1]	0.4 ± 0.8	0 [0–1]	0.852
OHIP 4	0.1 ± 0.4	0 [0–0]	0.1 ± 0.4	0 [0–0]	0.485
OHIP 5	0.1 ± 0.4	0 [0–0]	0.1 ± 0.4	0 [0–0]	0.930
OHIP Total Score	1.5 ± 2.0	1 [0–2]	1.2 ± 1.9	0 [0–2]	0.020

*OHIP* Oral Health Impact Profile, *SD* standard deviation

Of the study participants, 90.8% stated that they had a good or very good general health, but only 66.9% rated their oral health being good or very good. Regarding general health, this observation was largely shared when the parents answered the question regarding general health (98.6%). However, the parents rated the oral health status better (81.4%) than the children themselves.

## Discussion

Compared to adults, the assessment of health-related quality of life in children and adolescents represents a long-neglected topic, which has, however, increasingly moved into the focus of health research in recent times and is also gaining importance at the municipal and national level with regard to urgent questions of disease prevention and health promotion [17]. There is also a growing interest in the relationship between malocclusion or orthodontic treatment need and OHRQoL. Since malocclusion can be observed differently by different patients, it is essential to understand its impact from the patients’ perspective [2].

Current literature suggests that children and young people perceive an impact of malocclusions on OHRQoL [18, 19]: malocclusion is linked to decreased OHRQoL. Thereby, the most frequently applied instrument that is used to measure the impact of malocclusions on OHRQoL in children and adolescents is the Child Perception Questionnaire (CPQ). The CPQ was specifically developed for younger age groups (6–14 years) [20, 21]. The present study took another approach for assessing OHRQoL by using the OHIP. The reason for this is that the study is designed in such a way that the study participants will be re-examined in 2030 when they are 17 and 18 years old (module kfo‑6.2). Therefore, an instrument was chosen that can be used in adults as well as in children to be able to assess the disease impact over a longer time. In contrast to the CPQ, the OHIP—which was originally developed for adults—has already been applied in adolescent populations to measure OHRQoL regarding tooth avulsion and caries [13, 14]. Further studies conducted in Nigeria, Brazil, and India also used a short form of the OHIP in children to evaluate malocclusion and its impact on quality of life [22–24]. In the present study, the 5‑item version of the OHIP was used to measure OHRQoL. In general, the OHIP is a well-validated and internationally widely used questionnaire which has been adapted to many cultural settings [25]. The OHIP‑5 is the shortest version of the original questionnaire that started with 49 questions. Other short forms also exist (20-, 19-, 14-item versions). The 5‑item version reduces the number of items to 10% of those of the original instrument, it was designed to capture 90% of the information contained in the OHIP-49 summary score with a minimum number of items [26], making it an attractive tool for efficient OHRQoL measurement. Shorter instruments reduce the burden of patients and especially for children, which allows better focusing of their attention.

Recent reviews have shown that malocclusion affects OHRQoL [18, 19, 27], although levels appear to be low [18, 27]. Nevertheless, Alrashed et al. [19] found that the more severe the malocclusion is, the more it is associated with worse quality of life in terms of the psychosocial aspects and some physical aspects of OHRQoL [27]. It can impair quality of life by affecting function, appearance, interpersonal relationships, socializing, self-esteem, and psychological well-being. Current data also suggest that the effect of malocclusions on OHRQoL is modified by the age of the children and their cultural environment [18]. For Germany, data on the impact of malocclusion on OHRQoL are available for 11- to 14-year-old children. Bekes et al. [17] recruited children in a regional sample (Wernigerode, Saxony–Anhalt, Germany) during the annual dental public health examination. OHRQoL was measured using the German version of the CPQ. It was found that summary score differences in children with and without malocclusion were present and statistically significant (*p* = 0.0001).

Our study supports these findings. It could be shown that children aged 8 and 9 years with orthodontic treatment need had a significant higher OHIP score (1.5 ± 2.0) than children with no need for treatment (1.2 ± 1.9). The OHIP item representing the functional dimension (“difficulty chewing food”) also showed a significant difference in the mean score (0.4 ± 0.8 vs. 0.3 ± 0.8; *p* = 0.011). Moreover, children with pain aching tended to have a higher need for care. However, it should be mentioned that the observed level of impairment was low. In a recent review, Alrashed et al. [19] found that the impact of OHRQoL was 0.77 times lower for children with malocclusion than for those without malocclusion (SOR (standardized odds ratio) = 0.77, 95% confidence interval [CI] 0.46–1.30). We were not able to confirm these findings.

The present study has several strengths. One major strength is the representativeness with regard to the population of 8 and 9 year olds in Germany. The relatively high response rate of 40.6% and the number of 705 cases allow valid conclusions to be drawn about oral health in relation to orthodontic anomalies and its impact on OHRQoL. So far, the association of malocclusion and OHRQOL has mainly been evaluated in cross-sectional studies [18]. This study provides national representative data for Germany. No regional sample was used. Another strength of the study is the objectifiability of the orthodontic diagnosis and the classification of the need for treatment or no need for treatment. It was possible to measure the digital models of the jaws captured by intraoral scanner several times if needed. A further strength can be seen in the use of an OHRQoL instrument (OHIP) that has proven over years to have sound psychometric properties and is internationally accepted. On the other hand, one limitation might be that the OHIP has not been applied on a regular basis in studies in younger children up to date.

With the availability of national norm data for German children aged 8 and 9 years, broader application possibilities of the OHIP‑5 open up. The data presented can now be used for other studies in Germany that deal with OHRQoL in children in this age group and use the OHIP‑5 as OHRQoL instrument. In this way, children with different oral problems as well as different therapy variants can be evaluated with reference to our data from the population-representative sample.

## Conclusion

This study suggests that there is an association between orthodontic treatment need and poor oral health-related quality of life (OHRQoL).

## Footnotes

999 The BIK regions and interdependency areas are a nationwide spatial classification system in Germany that defines the urban-rural relationships at the municipal level for metropolitan areas, urban regions, middle and sub centers.
